# Sudden cardiac death in the young (5-39 years) in the canton of Vaud, Switzerland

**DOI:** 10.1186/1471-2261-14-140

**Published:** 2014-10-07

**Authors:** Fanny Hofer, Florence Fellmann, Jürg Schläpfer, Katarzyna Michaud

**Affiliations:** Faculty of biology and medicine, University of Lausanne (UNIL), Lausanne, Switzerland; University Hospital of Lausanne, Service of Medical Genetics, 1011 Lausanne, Switzerland; Department of Cardiology, University Hospital of Lausanne, 1011 Lausanne, Switzerland; University Center of Legal Medicine, Lausanne and Geneva, Rue du Bugnon 21, 1011 Lausanne, Switzerland

**Keywords:** Sudden cardiac death, Autopsy rate, Death certification

## Abstract

**Background:**

Sudden cardiac death (SCD) among the young is a rare and devastating event, but its exact incidence in many countries remains unknown. An autopsy is recommended in every case because some of the cardiac pathologies may have a genetic origin, which can have an impact on the living family members. The aims of this retrospective study completed in the canton of Vaud, Switzerland were to determine both the incidence of SCD and the autopsy rate for individuals from 5 to 39 years of age.

**Methods:**

The study was conducted from 2000 to 2007 on the basis of official statistics and analysis of the International Classification of Diseases codes for potential SCDs and other deaths that might have been due to cardiac disease.

**Results:**

During the 8 year study period there was an average of 292′546 persons aged 5-39 and there were a total of 1122 deaths, certified as potential SCDs in 3.6% of cases. The calculated incidence is 1.71/100′000 person-years (2.73 for men and 0.69 for women). If all possible cases of SCD (unexplained deaths, drowning, traffic accidents, etc.) are included, the incidence increases to 13.67/100′000 person-years. However, the quality of the officially available data was insufficient to provide an accurate incidence of SCD as well as autopsy rates. The presumed autopsy rate of sudden deaths classified as diseases of the circulatory system is 47.5%. For deaths of unknown cause (11.1% of the deaths), the autopsy was conducted in 13.7% of the cases according to codified data.

**Conclusions:**

The incidence of presumed SCD in the canton of Vaud, Switzerland, is comparable to the data published in the literature for other geographic regions but may be underestimated as it does not take into account other potential SCDs, as unexplained deaths. Increasing the autopsy rate of SCD in the young, better management of information obtained from autopsies as well developing of structured registry could improve the reliability of the statistical data, optimize the diagnostic procedures, and the preventive measures for the family members.

## Background

According to the World Health Organization (WHO), cardiovascular diseases are the leading cause of mortality in the world, accounting for 30% of deaths in 2008 [1]. According to the Swiss Federal Statistical Office (FSO), 33.8% of the 62′091 deaths registered in 2012 were of cardiovascular origin [2]. The rate of cardiovascular mortality increases with age, but cardiac deaths can also concern younger populations [3].

Approximately half of the cardiac deaths in young individuals are sudden, and often occur in individuals considered to be in good health [4, 5]. Up to 53% of the sudden deaths in children, teenagers and young adults remain unexplained, despite performing a complete autopsy; these deaths are often considered to be arrhythmic in nature, resulting from inherited channelopathies [6, 7]. Most of these disorders cannot be detected through a regular autopsy, but only by performing post-mortem genetic analyses, a.k.a. molecular autopsies or screening the first-degree relatives for inherited cardiovascular diseases [6, 8–13]. The identification of mutation carriers among the living family members allows for the prevention of potential premature deaths through the establishment of appropriate preventative measures [14].

The exact incidence of sudden death in young individuals remains unclear. According to the literature, the incidence of sudden death for individuals under 40 years of age is between 0.7 and 6.2/100′000 person-years [5, 15–24]. The incidence of SCD in Switzerland is not currently known, and there are only few data available on this matter in central Europe.

The Council of Europe recommends that the cause of death be established if at all possible and strongly encourages the use of autopsy. An autopsy plays an essential role in determining the cause of death and allows for its verification in an objective way. It also enables the documentation of precise demographic statistical data.

The aim of the present work is to establish the incidence of SCD between 2000 and 2007 in the canton of Vaud, Switzerland among individuals from 5 to 39 years of age on the basis of statistical data. Additional aims are to evaluate the autopsy rate in this population, and to determine the percentage of autopsies, which confirmed the diagnoses of SCD.

## Methods

### General considerations

The Canton of Vaud is one of the 26 Swiss cantons. According to Swiss Statistics, in 2012 the Canton of Vaud had a population of 734′356 inhabitants representing 9.1% of the Swiss population (8′039′060). The average population of individuals who aged between 5 and 39 years between 2000 and 2007 was of 292′546 inhabitants. In the canton of Vaud, the general practitioner who completes a death certificate has to determine if the death was natural, violent or undetermined. If the death is determined to be violent or undetermined, a medico-legal investigation is usually undertaken but does not systematically include an autopsy. A forensic autopsy is performed if the appropriate authorities deem it necessary. Familial consent is not required for a forensic autopsy. If the death is determined to be due to natural causes, a clinical autopsy can be performed with the consent of the living relatives. Several weeks after the death, the general practitioner who signed the death certificate is contacted by the Swiss Statistics Office and is asked to complete the medical certificate (page 53 of these recommendations) concerning the cause of death and any existing concomitant diseases [25]. Among others, the following questions are asked: 1) Was an autopsy performed? 2) If so, are the results available? and 3) If performed, did the autopsy confirm the previously determined cause of death? The general practitioner who signs the death certificate is not, however automatically informed if an autopsy was performed or not, especially in the forensic context. He or she rarely has access to forensic autopsy results, as they are usually transmitted only to judicial authorities. This being the case, the forensic pathologist who performed the autopsy is not automatically questioned about the results of the post-mortem investigations. At present the autopsy results are not required for statistical purposes, however, their integration into the statistical analyses is strongly encouraged as noted in the recommendations mentioned above, “if possible, the results of autopsy should be awaited before validation of the cause of death”.

### Data

This retrospective study relied on the gathering of statistical data from both the Cantonal Statistical Research and Information Service (SCRIS) and the University Institute of Social and Preventive Medicine (IUMSP) in the canton of Vaud, Switzerland from 2000 to 2007. The SCRIS provided general demographic statistical data on the total population, broken down into age groups and the cause of death. The IUMSP provided the information regarding the autopsies performed and was contacted by its website. The general statistical information was obtained from the website of Swiss Statistics.

### Methods

Among the deaths classified according to the ICD-10 code, cases which might correspond to SCD were extracted. Our methodology is based on previous studies with all its inherent limitations [5, 17, 20]. The first group included the cases classified as “diseases of the cardiovascular system” (Table 1). The second group included deaths whose origins were unclear or unknown, with the ICD-10 codes R96, R98, R99 and R09.2 (Table 2). The third group included deaths due to transport accidents, and those caused by drowning and submersion. When using ICD-10 codes with transport accidents, it is important to keep in mind that it is often difficult to determine whether the death was directly related to the vehicle’s driver or not. The incidence was established for the population of the defined age range between 2000 and 2007 in these 3 groups.

**Table 1:** **Deaths classified as diseases of the circulatory system**

		Age					
		15 - 19	20 - 24	25 - 29	30 - 34	35 - 39	15-39
**Men**	I21 Acute myocardial infarction	1		2		8	**11**
	I33 Acute and subacute endocarditis				1	1	**2**
	I42 Cardiomyopathy		2	1		2	**5**
	I46 Cardiac arrest	1			1	3	**5**
	I48 Atrial fibrillation and flutter			1			**1**
	I49 Other cardiac arrhythmias				2	1	**3**
	I50 Heart failure		1			1	**2**
	I51 Complications and ill-defined descriptions of heart disease	1	1			1	**3**
**Women**	I21 Acute myocardial infarction			1			**1**
	I40 Acute myocarditis	1	1	1			**3**
	I46 Cardiac arrest		1				**1**
	I49 Other cardiac arrhythmias		1			1	**2**
	I50 Heart failure				1		**1**
*Total*		*4*	*7*	*6*	*5*	*18*	*40*

Causes of death (number of cases) between 2000 and 2007 classified as diseases of the circulatory system that may potentially be sudden cardiac deaths among individuals from 5 to 39 years of age in the canton of Vaud, Switzerland. There were no deaths under the age of 15 (data received from SCRIS).

**Table 2:** **Deaths classified as “deaths with ill-defined and unknown causes”**

		Age							
		5-9	10-14	15-19	20-24	25-29	30-34	35-39	5-39
**Men**	R09.2 Respiratory arrest		1			1	5	9	16
	R96.0 Instantaneous death						1		1
	R98 Unattended death							2	2
	R99 Other ill-defined and unspecified causes of mortality	2	1	7	11	9	13	24	67
**Women**	R09.2 Respiratory arrest					1	2		3
	R98 Unattended death						1		1
	R99 Other ill-defined and unspecified causes of mortality		1	3	5	5	10	7	31
*Total*		*2*	*3*	*10*	*16*	*16*	*32*	*42*	*121*

Causes of death (numbers of cases) between 2000 and 2007 classified under to be due to “deaths with ill-defined and unknown causes” among individuals from 5 to 39 years in the canton of Vaud, Switzerland (data received from IUMSP).

The percentage of autopsies conducted was calculated using the data obtained from the IUMSP for all the deaths occurring in individuals from 5 to 39 years of age in the canton of Vaud between 2000 and 2007, as well as within each subgroup. We also analysed to what extent the autopsy results were used to update statistical data.

The protocol of this study was evaluated by the Cantonal (Vaud) Ethics Committee on research involving humans. No consent was requested as study was performed retrospectively on anonymous data, which was in accordance with the existing law.

## Results

**Figure 1:**
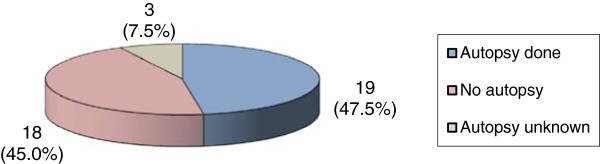
**Autopsies for potential sudden cardiac deaths.** Legend: Absolute Number and percent of autopsies for deaths considered as potential sudden cardiac deaths between 2000 and 2007 classified as “diseases of the circulatory system” among individuals from 5 to 39 years of age in the canton of Vaud, Switzerland (data received from IUMSP).

40 deaths were classified as presumed SCDs related to the cardiovascular system. The main cause of death in this group was “acute myocardial infarction”, consisting of 12 cases. This cause is predominant among men from 35 to 39 years of age (8 cases). It is followed by cardiac arrest (6 cases), other cardiac arrhythmias (5 cases) and cardiomyopathies (5 cases) (Table 1). An autopsy was conducted for 19 (47.5%) of the 40 deaths (Figure 1). An informative feedback concerning the autopsy results was available for only 4 cases (10%), allowing the confirmation of the initially certified cause of death (Table 3).

**Table 3:** **Numbers and percent of autopsies and diagnosis confirmation among the different groups of causes of death that may be at the origin of a sudden cardiac death, among people aged from 5 to 39 between 2000 and 2007, in the Vaud Canton of Switzerland**

	Cases	Autopsy act codified as performed	Autopsy act codified as not performed	Unknown if autopsy was performed	Cause of death confirmed after an autopsy
	N (%)				
All diseases of the circulatory system included	71 (6.3%)	34 (47.9%)	32 (45.1%)	5 (7.0%)	14 (41.2%)
Diseases of the circulatory system, potential sudden deaths	40 (3.6%)	19 (47.5%)	18 (45.0%)	3 (7.5%)	4 (21.1%)
Deaths by ill-defined and unknown causes	124 (11.1%)	17 (13.7%)	13 (10.5%)	94 (75.8%)	2 (11.8%)
Transport accidents	140 (12.5%)	39 (27.9%)	60 (42.9%)	41 (29.3%)	13 (33.3%)
Drowning/submersion	16 (1.4%)	11 (68.8%)	4 (25.0%)	1 (6.2%)	4 (36.4%)
*General/ all deaths*	*1122 (100%)*	*352 (31.4%)*	*507 (45.2%)*	*263 (23.4%)*	*130 (36.9%)*

This table presents a synthesis of the main statistical data. In the left column, percents were calculated according to the total number of deaths (1′122 deaths). In the four right columns, percents are related to the total number of cases from the corresponding category except the last column (percentages calculated for autopsied cases). The group “transport accidents” includes deaths that may derive from a cardiac cause, or a SCD resulting in loss of control of the vehicle. Drowning/submersions are normally classified in the group “other external causes of mortality”. The column on the very right includes those cases with feedback from the autopsy results, enabling confirmation of the cause of death (data received from IUMSP). It is possible that the percentage of autopsies with a confirmed cause of death could be higher if the autopsy results were neither not available nor considered during the codification process.

**Figure 2:**
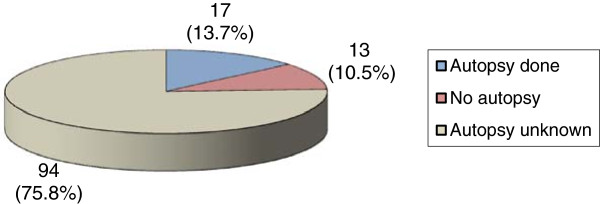
**Autopsies for unexplained deaths.** Legend: Absolute number and percent of autopsies for deaths between 2000 and 2007 classified as “ill-defined and unknown causes” among individuals from 5 to 39 years of in the canton of Vaud, Switzerland (data received from IUMSP).

The cause of death remained undetermined or unclear for 121 deaths that could possibly have been SCD (data received from SCRIS). These cases were more frequent in the 30 to 39 year age category (74/121; 61%) (Table 2). According to data received from IUMSP, an autopsy was performed in 13.7% of cases in this category of deaths and the autopsy results enabled the confirmation only for 2 deaths registered in the statistical data (Table 3). It was unknown if an autopsy was performed or not in 94 cases (75.8%) (Figure 2).

Table 3 lists also 140 selected cases that could have been potentially due to faintness or even SCD, leading to a loss of control of the vehicle. These deaths were followed by an autopsy in 39 cases (27.9%), which enabled confirmation of the cause of death in 13 cases (33.3% of autopsied cases). An autopsy was performed in 11 out of the 16 cases of drowning/submersions (68.8%), which confirmed the cause of death in 4 cases (36.4% of autopsied cases) (Table 3).

### Incidence of presumed SCD between 2000 and 2007 among individuals from 5 to 39 years

The calculated incidence of presumed SCD classified as diseases of the circulatory system is 1.71 per 100′000 person-years. The incidence is greater for males than females (2.73 and 0.69/100′000 person-years, respectively). It remains possible, however, that some cases of presumed SCD were misclassified as “deaths of ill-defined and unknown causes” (i.e. R96, R98, R99 and R09.2). If these ICD-10 codes are included in the calculation, the incidence of SCD increases to 7.01/100′000 person-years.

If the transport accidents and the drownings/submersions that could have potentially been of cardiac origin are included, even if this appears very unlikely, the presumed incidence increases even further to 13.67/100′000 person/years. As already explained in the methodology section, it is difficult to ascertain whether the death was directly related to the vehicle’s driver or not when using ICD-10 codes with transport accidents. It is also impossible to distinguish all possible primary causes of drowning on the basis of codified data. The presumed incidence varies by a factor 8 depending on which categories are considered. It is certain that all these deaths in the three categories are not SCDs, but it is difficult to determine to what extent.

## Discussion

The reliability of the incidence depends on the available statistical data, which is based on the information given by the physicians who declare the deaths. We noted that the quality of the officially given data was insufficient to provide an accurate incidence of SCD as well as autopsy rates. Therefore we use the term of “presumed” considering this limitation inherent to the current system of codification and to the retrospective nature of the study. The calculated incidence of presumed SCD for the deaths in the group related to cardiovascular disease is 1.71/100′000 person-years, and represents 3.6% of deaths among the studied population. These results are in accordance with those previously published in other countries [5, 15–24, 26–28]. A study conducted in England and in Wales showed an incidence of 1.8/100′000 person-years in a population between 1 and 34 years of age [20]. A Danish study calculated an incidence ranging from 1.9 to 2.8/100′000 person-years among people from 1 to 35 years of age, depending on whether or not the non-autopsied cases were included [5]. A study conducted in Ireland in a population from 15 to 35 years of age, found an incidence of 2.85/100′000 person-years [17]. A Canadian study estimated an incidence of 2.6/100′000 person-years among individuals from 2 to 40 years of age [21]. A prospective study conducted on young Italian athletes from the Veneto region came up with an incidence of 2.1/100′000 person-years. This same study estimated the incidence of non-athletes to be 0.7/100′000 person-years [15] (Table 4). To our knowledge, data on the incidence of SCD among the young in Switzerland has never been published, nor for other populations of central Europe. As a result, we cannot compare our results to those from other parts of Switzerland, nor with some of the bordering countries.

**Table 4:** **Literature data and our study**

Country (region)	Study	Age, years	Time period of the study	Incidence n/100′000	Type(s) of sudden deaths	Studied populations
Canada (Ontario)	Pilmer [21]	2-40	2008	2.6	SCD	General
Ireland	Margey [17]	15-35	2005-2007	2.85	SCD	General
Denmark	Winkel [5]	1-35	2000-2006	1.9-2.8	SCD	General
Greece (Epirus)	Fragkouli [16]	1-35	1998-2008	1.78	SCD	General
Ireland	Morris [19]	14-35	2005	3.18	SCD	General
Netherlands	Vaartjes [23]	1-40	1996-2006	2.07	SD	General
USA	Maron [27]	8-39	1980-2006	0.61	SCD	Athletes
England and Wales	Papadakis [20]	1-34	2002-2005	1.8	SCD	General
USA	Eckart [26]	18-35	1977-2001	13	Non-traumatic SD	Military
Spain (Bizkaia)	Morentin [18]	1-35	1991-1998	2.4	SD	General
Italy (Veneto)	Corrado [15]	12-35	1979-1999	0.7-2.1	SCD	Athletes vs non-athletes
Sweden	Wisten [28]	15-35	1992-1999	0.93	SCD	General
England	Wren [24]	1-20	1985–1994	3.3	SD	General
USA (Minnesota)	Shen [22]	20-40	1960-1989	6.2	SD	General
Switzerland (Vaud Canton)	present study	5-39	2000-2007	1.71	SCD	General

*Incidence of sudden death among populations under 40 years old, SD (sudden death); SCD (sudden cardiac death).*

We acknowledge the difficulty in determining a precise incidence of SCD due to the necessity to isolate, based on the ICD-10 codes, the cases of interest from all the other deaths caused by diseases of the circulatory system [5, 20]. Furthermore, the ICD-10 codes do not indicate the continuity or the temporal links of the events leading up to death, leaving a lot of room for interpretation. A substantial number of deaths (11%) were not classified under any defined cause (i.e. R96, R98, R99 and R09.2). This group could include cases of SCD, intoxications or other deaths that do not leave any visible trace for the practitioner conducting the summary examination upon declaring the death. The incidence based only on cases classified as cardiovascular deaths, excludes situations where a cardiac faintness could be at the origin of the fatal chain of events resulting in death, such as an epileptic crisis, drowning, or car accident [20, 29, 30].

The global autopsy rate within the selected age group is 31.4%. For deaths related to diseases of the circulatory system that could potentially be a SCD, the autopsy percentage increases to 47.5%. This autopsy rate in the canton of Vaud, Switzerland is low compared to that of other countries. This rate is astonishing, as the recommendations of the Council of Europe specify that an autopsy should be performed in every case of unexpected sudden death [31]. For instance, in Denmark, 75% of sudden unexpected deaths among people from 1 to 35 years of age undergo an autopsy and this percentage was thought to be far from optimal when the aim is to establish adequate statistics of the causes of death [5]. In Ireland, this rate is 86.3% for SCDs among those from 15 to 35 years of age [17]. In our population, the autopsy was conducted in only 13.7% of the cases with ill-defined and unknown causes, which represent 11% of all the deaths. It was difficult, if not impossible, to determine the influence of the autopsy on the final classification of the cause of death. Thus, there is a substantial proportion of deaths of unknown cause with uncertain autopsy results. This group likely includes unrecognized SCDs, which will not undergo further investigation nor contribute to the data in the appropriate statistical group. We were surprised to find that the autopsy results often remained undisclosed, and were not included in the larger group of statistical data. The diagnosis was confirmed in only a relatively low proportion of cases (11.6% of all cases and 36.9% of autopsied cases). When the diagnosis remained questionable or unconfirmed, it was impossible to determine if the cause of death was modified according to the post-mortem examination in the official published statistics.

The information obtained from the death certificates, which contains the basic information for the statistical analysis concerning mortality [9, 32], differs between countries. In Denmark, the death certificate must be filled out by several individuals and contains a specific field for complementary information, such as circumstances of death, testimonies from witnesses and relatives, former medical conditions, a description of the external body examination and the preliminary conclusions before autopsy [5]. Despite the relatively high proportion of autopsies and the complementary information reported on the death certificate, the Danish register of statistics on the causes of death was considered insufficiently reliable by the authors of the study. They concluded that 24% of SCDs or unexplained sudden deaths were misclassified. Our study confirms that the use of death certificates in the statistical analysis of the causes of mortality is far from ideal as was already suggested by other authors [9, 32, 33]. It is of the utmost importance to find solutions to facilitate the coordination between the various individuals involved in the transmission of information, such as improving the feedback system and instructing medical students on how to correctly fill out death certificates [9] or developing structured registry of SCD in the young. The death certificate should be improved in order to provide more complete information about the circumstances of death, and to allow for easier and more representative codification. The solution will not be simple as it implicates several domains, such as medicinal, judicial and political. If the present situation is not improved the value of the published statistics on the causes of mortality will remain scientifically unreliable. Moreover, considering the genetic origin of many of SCDs, prevention of further deaths in family members without performing an autopsy will remain shackled.

## Conclusions

This study is the first in Switzerland informing about the magnitude of SCD. The incidence of presumed SCD in the canton of Vaud, is comparable to the data published in the literature for other geographic regions but may be underestimated as it does not take into account other potential SCDs, as unexplained deaths. Better management of information obtained from autopsies and death certifications as well increasing the autopsy rate, developing structured registry of SCD in the young could improve the reliability of the statistical data. Autopsy findings are important not only to correctly codify the cause of death but also to optimize the diagnostic procedures and the preventive measures for the family members, considering the frequency of genetic diseases associated with sudden cardiac death.

### Limitations

The limitations of the study are inherent to the retrospective review of data from our regional registries and low autopsy rate. However, the autopsy rate may be underestimated considering that the information concerning to whether an autopsy was performed or not, was unavailable in 23.4% of cases. The available anonymous data does not allow for the distinction between SCD and other cardiovascular deaths. For this reason the term “presumed SCD” has been chosen. Similar limitations have been reported in other published studies of this nature [9, 32, 33].

We think that our first evaluation of the magnitude of SCD in the canton of Vaud, Switzerland will be helpful to stimulate debate concerning the clinical implications of low autopsy rate in the young as of a lack of correlation between autopsy data and official statistics. We hope that our study will encourage further research in order to improve the situation.

## Abbreviations

SCD: Sudden cardiac death
SCRIS: Cantonal statistical research and information service
IUMSP: University Institute of Social and Preventive Medicine
FSO: Swiss Federal Statistical Office.
